# Strengthening our resolve for primary health care

**DOI:** 10.2471/BLT.20.279489

**Published:** 2020-11-01

**Authors:** Tedros Adhanom Ghebreyesus

**Affiliations:** a World Health Organization, Avenue Appia 20, 1211 Geneva 27, Switzerland.

The 1978 Declaration of Alma-Ata marked a paradigm shift in the global approach to health. The concept of primary health care moved from the traditional hospital-centred, biomedical approach to one focused on meeting the health needs of communities and addressing health inequities.

The goal of primary health care is to provide a person-centred, holistic approach to health, focused on prevention and the continuum of well-being. Primary health care includes those primary care services typically delivered at clinics and hospitals, but also emphasizes addressing the determinants of health to promote health, prevent disease and avoid or delay the need for curative services.

The 2018 Declaration of Astana,^1^ with its commitment to health for all, further operationalized primary health care as central to the achievement of universal health coverage (UHC) and the health-related sustainable development goals (SDGs). This expanded concept of primary health care was reinforced the following year with the publication of the UHC global monitoring report, *Primary health care on the road to universal health coverage*.^2^

The Declaration of Astana defines the three core pillars of primary health care as (i) primary care and essential public health functions as the core of integrated health services; (ii) multisectoral policy and action; and (iii) empowered people and communities.^1^ In practice, this means that primary health care provides the range of health services needed throughout the life course, including promotion, prevention, treatment, rehabilitation and palliative care.

Primary health care is revolutionary in its embrace and demands for a multisectoral approach that goes beyond the health sector, linking individual care to population health and health determinants. Such an approach also addresses the importance of equity, social participation and social justice in the achievement of health for all.

The coronavirus disease 2019 (COVID-19) pandemic and its effects on health outcomes globally has further highlighted the importance of primary health care. Leadership, the strength of health systems and the public’s confidence in those systems have been determining factors in the quality of the response to the pandemic. Emergency response measures and services, including treatment, testing and contact tracing, rely on a strong public health infrastructure and a workforce that has earned the trust of their communities. COVID-19 has shown that trust, solidarity and cooperation between people are essential elements in the response to regional and global health emergencies. The pandemic has further underscored the essential primary health-care principle that protecting and promoting health requires a whole-of-government and whole-of-society approach.

Nonetheless, the broad approach that is integral to primary health care remains neglected and underfunded. Globally, investments in secondary and tertiary care tend to outstrip those in primary health care.^3^ This failure to focus on and fund prevention misses a huge opportunity to provide effective, efficient and equitable means to address the main causes and risk factors of poor health.^4^

Climate change and shifting disease patterns also provide an impetus for greater investments in primary health care,^5^ which is the foundation for making progress against all of the health-related SDG targets, including communicable and noncommunicable diseases, maternal, newborn, child and adolescent health, and healthy ageing.

Significant challenges remain. Moving to implementation requires addressing major hurdles, including measuring primary health-care implementation in low- and middle-income countries. Addressing this gap is critical for the accountability and responsiveness that are key to improving patient satisfaction and the quality of services.^6^ We also need better and more evidence and guidance to enable us to address the financial, infrastructural, political and technical challenges of policy implementation. We should build on the strong evidence base from low- and middle-income countries in areas such as financing and health workforce to address other areas of implementation.^7^ To do so, we need context-specific health policy and systems research, tailored to locally defined challenges.

The World Health Organization (WHO)’s commitment to primary health care has never been stronger. To support Member States to strengthen primary health care, we have recently established a special programme that brings together expertise across the various technical areas of WHO and at the country, regional and headquarter levels. Going beyond the health ministry will require national coordination and work across sectors such as water and sanitation, urban development and design, commercial regulation and education. These multisectoral efforts to strengthen primary health care must have equity at their core, and be a solid foundation for pandemic responses and emergency preparedness.

Strengthening primary health care will help us respond to future challenges. Ultimately, primary health care is an investment in a healthier, safer, fairer and more sustainable future.
